# Comparison of automated segmentation techniques for magnetic resonance images of the prostate

**DOI:** 10.1186/s12880-023-00974-y

**Published:** 2023-02-11

**Authors:** Lars Johannes Isaksson, Matteo Pepa, Paul Summers, Mattia Zaffaroni, Maria Giulia Vincini, Giulia Corrao, Giovanni Carlo Mazzola, Marco Rotondi, Giuliana Lo Presti, Sara Raimondi, Sara Gandini, Stefania Volpe, Zaharudin Haron, Sarah Alessi, Paola Pricolo, Francesco Alessandro Mistretta, Stefano Luzzago, Federica Cattani, Gennaro Musi, Ottavio De Cobelli, Marta Cremonesi, Roberto Orecchia, Giulia Marvaso, Giuseppe Petralia, Barbara Alicja Jereczek-Fossa

**Affiliations:** 1 Department of Radiation Oncology, IEO European Institute of Oncology IRCCS, Milan, Italy; 2 Division of Radiology, IEO European Institute of Oncology IRCCS, Milan, Italy; 3 Department of Oncology and Hemato-Oncology, University of Milan, Milan, Italy; 4 Molecular and Pharmaco-Epidemiology Unit, Department of Experimental Oncology, IEO European Institute of Oncology IRCCS, Milan, Italy; 5 Radiology Department, National Cancer Institute, Putrajaya, Malaysia; 6 Division of Urology, IEO European Institute of Oncology IRCCS, Milan, Italy; 7 Medical Physics Unit, IEO European Institute of Oncology IRCCS, Milan, Italy; 8 Radiation Research Unit, IEO European Institute of Oncology IRCCS, Milan, Italy; 9 Scientific Direction, IEO European Institute of Oncology IRCCS, Milan, Italy; 10 Precision Imaging and Research Unit, Department of Medical Imaging and Radiation Sciences, IEO European Institute of Oncology IRCCS, Milan, Italy

**Keywords:** Segmentation, Prostate, MRI, Deep learning, Medical image

## Abstract

**Background:**

Contouring of anatomical regions is a crucial step in the medical workflow and is both time-consuming and prone to intra- and inter-observer variability. This study compares different strategies for automatic segmentation of the prostate in T2-weighted MRIs.

**Methods:**

This study included 100 patients diagnosed with prostate adenocarcinoma who had undergone multi-parametric MRI and prostatectomy. From the T2-weighted MR images, ground truth segmentation masks were established by consensus from two expert radiologists. The prostate was then automatically contoured with six different methods: (1) a multi-atlas algorithm, (2) a proprietary algorithm in the Syngo.Via medical imaging software, and four deep learning models: (3) a V-net trained from scratch, (4) a pre-trained 2D U-net, (5) a GAN extension of the 2D U-net, and (6) a segmentation-adapted EfficientDet architecture. The resulting segmentations were compared and scored against the ground truth masks with one 70/30 and one 50/50 train/test data split. We also analyzed the association between segmentation performance and clinical variables.

**Results:**

The best performing method was the adapted EfficientDet (model 6), achieving a mean Dice coefficient of 0.914, a mean absolute volume difference of 5.9%, a mean surface distance (MSD) of 1.93 pixels, and a mean 95th percentile Hausdorff distance of 3.77 pixels. The deep learning models were less prone to serious errors (0.854 minimum Dice and 4.02 maximum MSD), and no significant relationship was found between segmentation performance and clinical variables.

**Conclusions:**

Deep learning-based segmentation techniques can consistently achieve Dice coefficients of 0.9 or above with as few as 50 training patients, regardless of architectural archetype. The atlas-based and Syngo.via methods found in commercial clinical software performed significantly worse (0.855$-$0.887 Dice).

## Background

Segmenting anatomical regions of interest (ROIs) such as organs and lesions in medical images is a standard procedure in many medical workflows. In radiotherapy, accurate segmentation of both lesions and surrounding organs in MRI images is needed to calculate the radiation dose and estimate the risk of normal tissue complications. In prostate fusion biopsy, segmentation is crucial to guide the collection of the cancerous specimen. Typically, the ROIs are manually or semi-automatically hand-drawn by trained medical personnel in treatment planning systems, but may suffer from poor reproducibility and/or accuracy [1–4]. In addition, since the procedure is time-consuming and requires trained experts, it can pose a burden on the medical workflow, and therefore, it is of great interest to develop automatic segmentation techniques that can be integrated into medical practice. The growing interest in computer-aided prediction models such as radiomics, where predefined mathematical features are calculated from the ROIs, has further increased the need for accurate automatic segmentation techniques.

The current state-of-the-art models for automatic image segmentation are predominantly deep learning (DL)-based. These are typically characterized by heavy use of convolutional operations, which enables the models to successively and automatically extract relevant features at different resolutions and locations in the images. The most famous and widely applied such model is the U-net [5], which was the first DL architecture to achieve widespread success in the field of medical image segmentation. Virtually all current best performing DL-based models are evolutions of the U-net that have incorporated various modifications, such as attention mechanisms [6, 7] or bottleneck convolutions [8]. However, instead of seeing common trends appear, increasingly different approaches and models are seeing use. Moreover, even for a given segmentation task, reports on different segmentation techniques are hard to compare, since they typically use different data sets, and suitable public medical image data sets for benchmarking are often not available or readily accessible.

Although advancements are continuously being made within the field, the automatic segmentation methods employed in common treatment planning and patient monitoring software tend to lag behind relative to the most recent results in the literature. This may be part of the reason manual segmentation is still the method of choice in most clinics. At present, it is not well known how the segmentation algorithms in proprietary medical software compare against recent DL advances.

To facilitate the integration of automatic segmentation models into clinical practice, it will be important to analyze how and when automatic segmentation is appropriate. For instance, patients with exceptionally large or small prostate volume may be particularly hard for them to segment, which would call for human intervention. Other factors such as age or the severity of the patient’s condition may also play an important role. It is also critical to try to gauge how much patient data is needed to train the segmentation models to an acceptable standard so that clinics can readily devote the appropriate amount of resources. In the current autosegmentation literature, these types of considerations are usually left out.

In this work, we compare four automatic DL segmentation models on a single data set consisting of 100 T2-weighted MRI scans of prostate cancer patients. We also benchmark the DL models against multi-atlas segmentation and a proprietary segmentation algorithm in the Syngo.via clinical imaging software developed by Siemens Healthineers. The models were chosen to cover some of the most essential training strategies (2D vs. 3D, training from scratch vs. transfer learning) and architectures (V-net, GANs, EfficientNet). Our main contributions can be summarized as follows:

- We compare the segmentation performance of four common DL segmentation models and training strategies: a V-net (a 3D evolution of the popular U-net), a transfer learning approach with weights pretrained on the ImageNet dataset, a generative adversarial network (GAN), and a 3D version of the EfficientDetB0 architecture adapted for segmentation.
- We benchmark our models against two retail medical software tools: Syngo.via (a proprietary DL-based algorithm developed by Siemens Healthineers) and Raystation 9B (a multi-atlas based algorithm from RaySearch Laboratories).
- We investigate whether clinical patient parameters such as risk class, prostate volume, and prostate specific antigen (PSA) levels have any impact on the segmentation performance. Such data could help institutions and clinics with defining personalized protocols to indicate whether or not specific patients are suitable for automatic segmentation.
- Lastly, we repeat our experiments with different amounts of training data (70 patients in training and 30 held out, as well as 50 in training and 50 held out) in order to evaluate how impactful and important the size of the data set is when training clinical segmentation models.

## Methods

### Data set

This study was conducted on a set of 100 T2-weighted MRI scans of the prostate from cancer patients at IEO European Institute of Oncology IRCCS, Milan, Italy. All patients gave their consent for use of their data for research and educational purposes, and the use of the data was approved by the local Ethical Committee, which waived the requirement for further consent specific to this study. The images were acquired using a 1.5 T scanner (slice thickness 3.0$-$3.6 mm, slice gap 0.3 mm, pixel spacing 0.59 $\times$ 0.59 mm, echo time 118 ms, and repetition time 3780 ms). For every image, a ground truth segmentation was established by consensus from two experienced radiologists with more than five years of experience.

The MRI volumes were resampled to a common size of $320\times 320 \times 28$ using bi-linear interpolation in the three cases where the image was larger than $320 \times 320$, and zero padding where there were fewer than 28 slices. Each image was corrected with the N4 bias field correction algorithm using the SimpleITK 2.0.2 python package with default parameters. Within each image, the intensities were clipped to the 0th and 98th percentile interval, and subsequently standardized to the [0, 4033] range^1^ (this is akin to a histogram normalization without landmark points, and is a crucial step for quantitative MRI analysis [9, 10]). For the DL-based applications, each image was also normalized to zero mean and unit variance.

The images were randomly divided into two different training and testing sets on which the models were evaluated: one 70/30 split and one 50/50 split. This allowed us to validate our results with repeated measurements and to test the reliability of the models in terms of training set size. In order to not let particular clinical characteristics confound the results of the study, we checked that the distribution of prostate volume and extraprostatic extension were similar within the training and test sets using the Mann-Whitney U-test.

### Experiments

We compared the performance of six different automatic segmentation models (described in detail in the following section):

1. A multi-atlas based algorithm in the Raystation 9B treatment planning software
2. A proprietary DL-based algorithm in the Syngo.Via medical image platform
3. A V-net, which is a 3D evolution of the popular U-net architecture.
4. A Transfer-learned U-net with a EfficientNetB0 [11] encoder with weights pre-trained on the Imagenet dataset.
5. A GAN extension of the transfer-learned network from point 4
6. A a version of the EfficientDet architecture modified for segmentation purposes [12].

Each model was trained and evaluated once on the 70/30 train/test-set split and once on the 50/50 split. The performance evaluation was based on the Dice score, absolute relative volume difference (ARVD), mean surface distance (MSD), and 95th-percentile Hausdorff distance (HD95) to the reference standard (ground truth). The scores are defined as follows:

The **Dice** coefficient is a measure of the overlap between two geometrical objects, defined by:

1 $$\begin{aligned} \text {Dice}(A,B) = \frac{2 | A \cap B |}{|A|+|B|} \end{aligned}$$

where *A* and *B* are the sets of pixels of the objects being compared. Dice coefficients range from 0 to 1, where 1 corresponds to perfectly overlapping objects, and 0 corresponds to any configuration where their intersection is zero.

**ARVD** is the absolute volume difference calculated relative to the volume of the ground truth (in this case *B*), defined by:

2 $$\begin{aligned} \text {ARVD}(A,B)=|\frac{V_A-V_B}{V_B}|. \end{aligned}$$

An ARVD of 0.3, therefore, means that the predicted prostate is either 30% larger or 30% smaller than the ground truth. A perfect score of 0 indicates that the objects have identical volume, but does not necessarily mean that the prostates are well aligned spatially.

**MSD**: The MSD (measured in pixels) between the surfaces $A_s$ and $B_s$ of *A* and *B*, is defined as

3 $$\begin{aligned} \text {MSD}(A,B) = \frac{\sum _{a \in A_s} \text{ d }(a, B_s) + \sum _{b \in B_s} \text{ d }(b, A_s)}{|A_s|+|B_s|} \end{aligned}$$

using the Euclidean distance from pixel $a\in A_s$ to surface $B_s$ given by $\text{ d }(a,B_s)=\min _{b\in B_s} ||a-b||$. The $|A_s|+|B_s|$ in the denominator is the total number of pixels in $A_s$ and $B_s$ combined (dividing by this sum results in an average distance). Since manual segmentations are drawn and evaluated on a slice-by-slice basis (and since the pixel spacing is spatially anisotropic), we calculated this quantity in 2D. This means, however, that the distance will be undefined in slices with at least one empty contour set. To avoid this, we redefine the empty contour as a single pixel in the center of the image.

**HD95**: the 95th percentile Hausdorff distance between two geometrical objects *A* and *B* is defined by:

4 $$\begin{aligned} \text {HD95}(A,B) = \max \left\{ \sup _{a\in A} \inf _{b\in B} \text {d}(a,b),\sup _{b\in B} \inf _{a\in A}\text {d}(a,b)\right\} \end{aligned}$$

where the supremum functions in this case return the 95th percentile values, making the metric more robust to irregularities. As with the MSD, we calculated this quantity on a slice-by-slice basis (i.e. in 2D).

The following analyses were performed on the segmentation results:

1. We compared the models in terms of their segmentation performance and variance. We also tested if the best-performing model was significantly outperforming the other models using the Wilcoxon signed-rank test.
2. We analyzed the performance of the methods in terms of the most poorly segmented patients. Since the segmentations are made on images from real patients, it is of great importance to not jeopardize any downstream implications resulting from unacceptable segmentations.
3. We tested if the methods systematically overestimate or underestimate the volume by calculating the signed relative volume difference and measuring its statistical deviation from unity (Wilcoxon signed-rank test).
4. In order to find potential confounding factors that may have a significant impact on the quality of the automatically generated contours, we looked at associations between the results and different clinical variables (age, prostate volume, iPSA, ISUP grade, ECE score, and PIRADS category). If found, this could serve to indicate whether a given prostate is suitable for automatic segmentation. It may also be useful for constructing better segmentation algorithms in the future. This analysis was made with the Kruskal-Wallis tests (for categorical variables), and Spearman correlation (for continuous variables). This analysis was only performed on the Dice and MSD performance metrics.
5. Lastly, we tested whether the increased training size in the 70/30 split significantly improved the performance of the best model compared to the 50/50 split. This can indicate whether datasets of this size are adequate to train a model, or if larger datasets are needed. The performance increase resulting from adding data will diminish at some point, and understanding this interplay will be important for future studies, especially because good-quality clinical data is hard to collect. This analysis was done with the Kruskal-Wallis test (since samples were of different size). As an extension of this analysis, we also performed a pair-wise Wilcoxon signed rank test on all patients in the intersection of the 70/30 and 50/50 test sets (a total of 20 patients).

Since the control of Type I error with multiple comparison corrections leads to an increase in Type II errors (and thus a reduction of the statistical power), no FDR corrections were applied in the above analyses. This reduces the probability of discarding potential associations in this explorative study (see [13] for further discussion).

### Segmentation models

#### Atlas (Raystation software)

Multi-atlas segmentation is a common automatic segmentation method built on ideas from deformable registration and coordinate transformation [14–16]. Even though they have recently fallen out of favor since the introduction of DL, they are still implemented in many treatment planning and picture archiving software, which makes them relatively accessible. The atlas method used herein is embedded in the RayStation 9B treatment planning system commonly used in radiation oncology. The implementation is based on the anatomically constrained deformation algorithm (ANACONDA), which combines image intensity data with anatomical information using contoured image sets. This solution requires the user to define atlases of images and contours, which the algorithm then uses to find a coordinate transform between the new image and the already segmented images in the atlas. If the atlas contains images similar to the new image, the coordinate registration will be more accurate, which will result in a more credible segmentation.

#### Siemens (Syngo.Via software)

The Syngo.Via medical image reading and post-processing software offers a built-in automatic segmentation method based on DL [17]. Partial details on its implementation have been published in relation to liver segmentation, but further information remains undisclosed to the public. Much of the appeal of this approach is that it is implemented in software that many clinicians are already familiar with, which increases its potential for integration and decreases the learning curve.

#### V-net

The basis of the V-net [18] segmentation model is its encoder-decoder architecture: an encoder first distills relevant information about the input image into representative features, then a decoder extracts useful information from these features into a desired structure—in this case, a binary segmentation matrix. The encoder and decoder are in turn built up from serially connected convolution blocks that operate at different resolutions by using intermediate upsampling and downsampling operations. The encoder and decoder are also connected horizontally by skip connections between each resolution. The primary modification in the V-net design was to use 3D convolutions for volumetric medical images, but it also incorporated residual convolution blocks and convolutional up- and down-sampling instead of pooling operations.

**Fig. 1:**
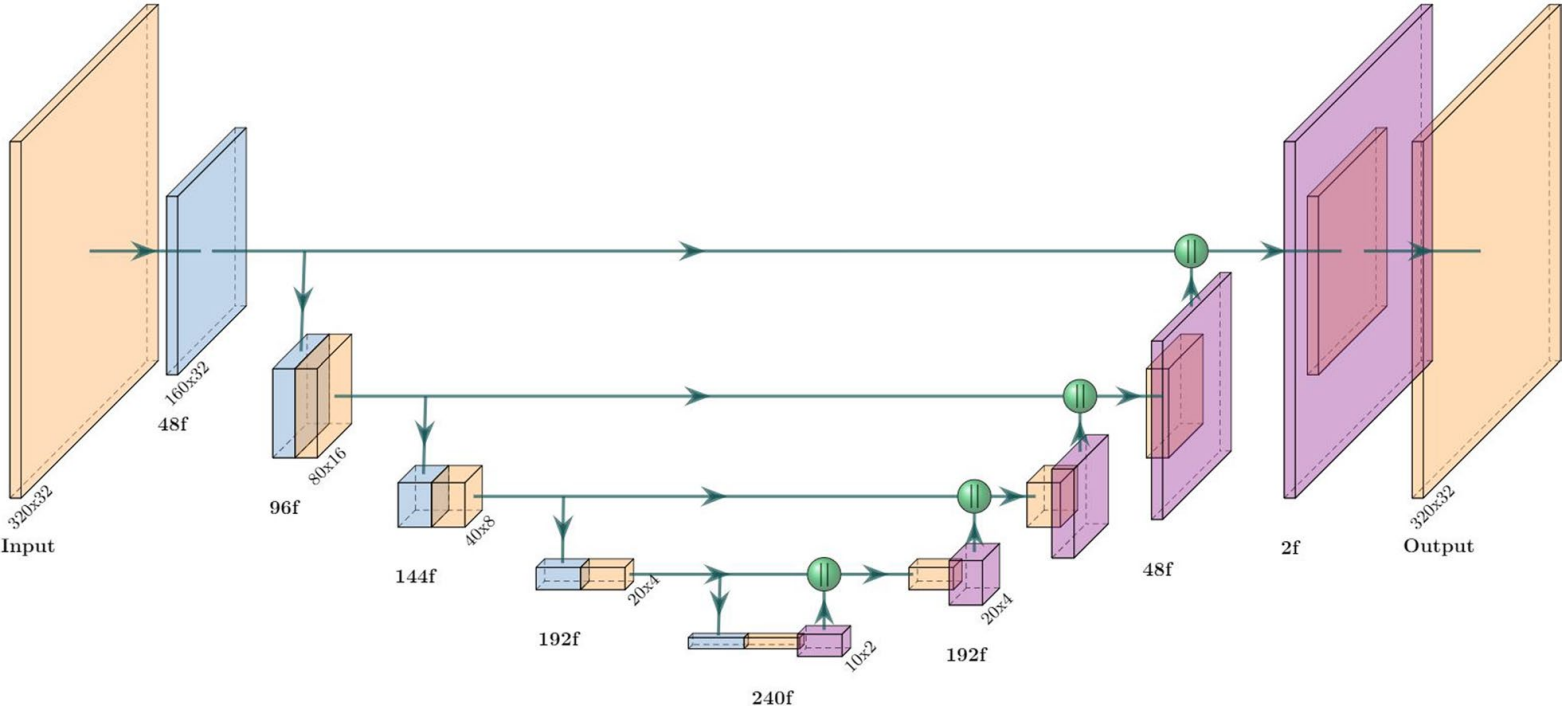
Architecture of our V-net segmentation network. Each block represents a residual convolution block (see Eq. 5); strided convolutions (downsampling) are blue and transposed convolutions (upsampling) are purple. The output resolution at each level is displayed and the number of filters/channels is displayed with the ‘f’ suffix. Green spheres represent concatenation. The last block performs 1x1x1 convolution with a sigmoid activation function

Our V-net implementation is summarized in Fig. 1. The residual convolutional blocks we used can be formalized as

5 $$\begin{aligned} \mathrm {ResConv( x ) =PReLU(Dropout(BatchNorm(Conv3D( x )))) + x } \end{aligned}$$

where PReLU is the parameterized rectified linear unit activation function [19]. We use non-spatial dropout with a rate of 0.5 and only apply it in the encoder. All the 3D convolutions used a $3\times 3\times 3$ kernel size apart form the transposed convolutions (used for upsampling) which used $2\times 2\times 2$. The first convolution is a single strided convolution (e.g. not a residual block) with 48 filters that downscales the resolution from $320\times 320\times 28$ to $160\times 160\times 28$. Every successive level adds (or subtracts in the decoder stage) another 48 filters apart from the last transposed convolution, which uses two filters. We also use concatenations for the horizontal connections. The output layer is a single $1\times 1\times 1$ 3D convolution with a sigmoid activation function.

#### Transfer learned U-net

Transfer learning (TL) is a common learning approach when data or resources are scarce or otherwise tainted (e.g. by poor quality). The idea is to apply pre-trained weights trained on a much larger dataset and/or a broader task in order to save computing resources and leverage the robustness of previously learned features. Hence, it’s a convenient choice in medical image analysis where data is often sparse.

**Fig. 2:**
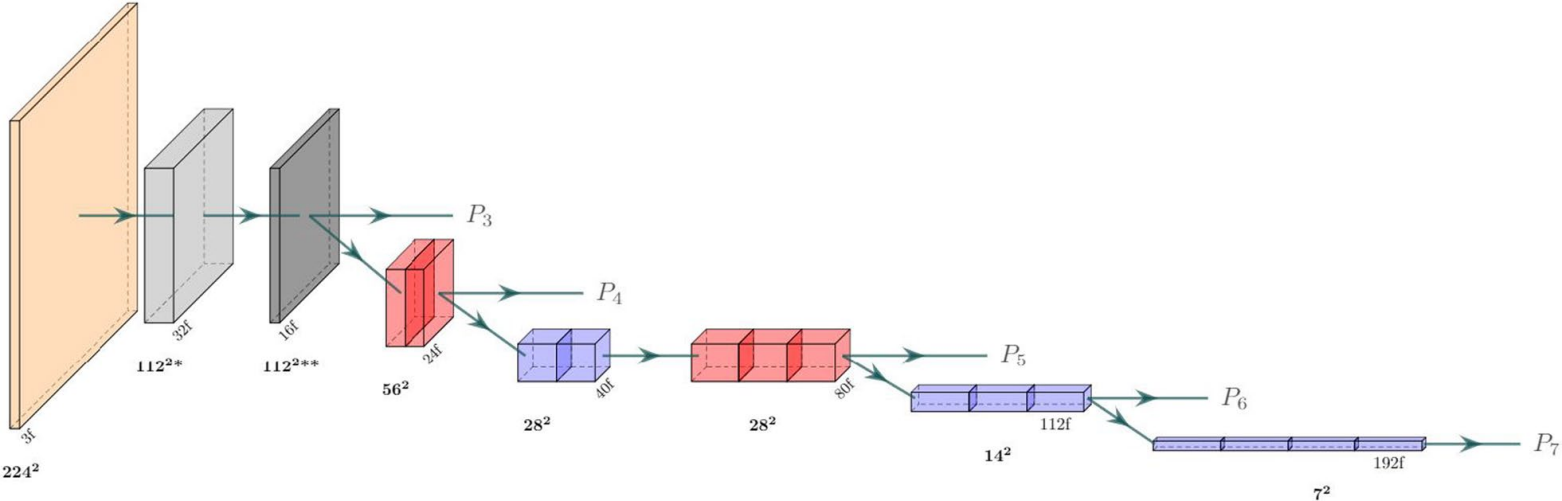
Architecture of the EfficientNetB0 backbone used in our transfer learning model. Blue and red blocks represent mobile inverted bottleneck convolution (MBConv) blocks (see Fig. 3), with a kernel size of 3x3 and 5x5, respectively. The light gray block represents a strided convolution followed by batch norm and a swish activation, while the dark gray block represents an MBConv block with expansion factor 1 and kernel size 3. The resolution at each level is displayed in bold and the number of filters (output channels) is displayed with the ‘f’ suffix

**Fig. 3:**
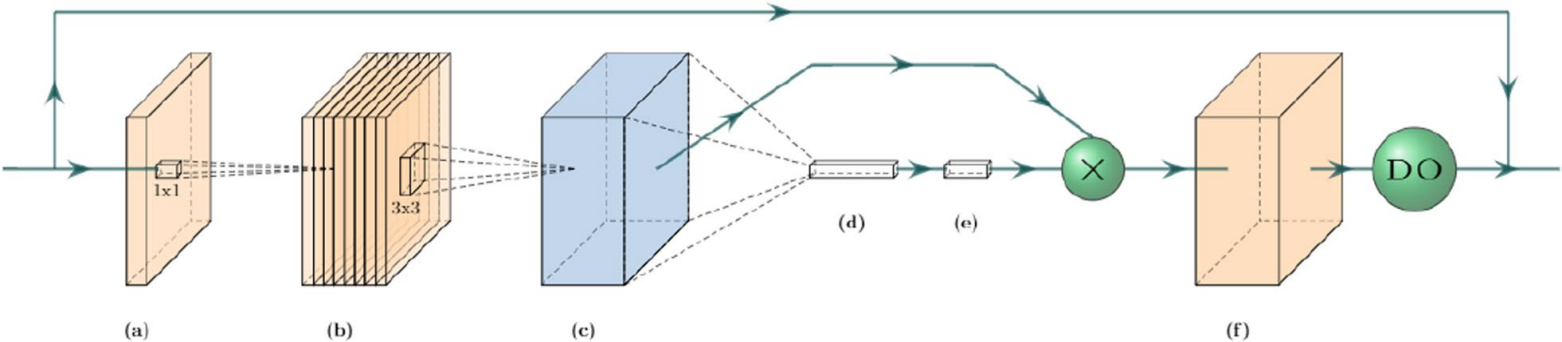
The mobile inverted bottleneck convolution (MBConv) block. (**a**) An initial 1x1 conv block expands the number of input channels according to the expansion factor hyper-parameter. (**b**) Depth-wise 3x3 conv block over channels. (**c**) Global average pooling shrinks the tensor along its spatial dimensions. (**d**, **e**) A squeeze conv (1x1 conv + swish) and an excitation conv (1x1 conv + sigmoid) first squeeze the channel dimension by a factor of 0.25, then expand it back to its original shape. The output is multiplied by the output tensor from step (**b**). (**f**) A final 1x1 conv block with a linear activation maps the tensor to the desired number of output channels, followed by a dropout layer for stochastic depth (dropout rate 0.2)

Our TL approach consisted of a U-net decoder stacked on top of an EfficientNetB0 encoder (Fig. 2) pre-trained on the 2012 ILSVRC ImageNet dataset for images classification. We chose the EfficientNetB0 backbone for its performance and efficiency; its authors demonstrated state-of-the-art performance on ImageNet while being up to 8.4x smaller and 6.1x faster than previous models. A key aspect of the EfficientNet architecture is that it processes the images and intermediate features with mobile inverted bottleneck convolution (MBConvs) blocks (see Fig. 3 for details). Since the ImageNet samples are 2D images, this architecture can also only process 2D inputs, which means that the implementation operates on a slice-by-slice basis. To implement this model, we used the Segmentation Models [20] python package.

To transform the $320\times 320\times 28$ MRI scans into the $224\times 224\times 3$ images required of the ImageNet weights, an initial $3\times 3\times 3$ convolution maps each slice into three-channelled images. They were then center-cropped into $224\times 224$. The final network output was constructed by concatenating and zero-padding them back to their original $320\times 320\times 28$ resolution. The number of filters in the decoder was set to {360, 288, 216, 144, 72} for levels {$P_7$, $P_6$, $P_5$, $P_4$, $P_3$}, and their kernel size was set to $4\times 4$. Other parameters were configured to match our U-net implementation.

#### Generative adversarial network (GAN)

GANs are a family of models where two network agents—one generator and one adversarial/critic/ discriminator—compete against each other with a shared objective function. The generator is trained to generate “fake” samples (in our case segmentation masks) with the aim of trying to fool the discriminator into thinking they are real, while the discriminator is trained to distinguish fake/generated samples from real ones. As the generator gets better at generating realistic-looking samples, the discriminator has a harder time identifying them as fake. And when the discriminator improves its discrimination performance, the generator needs to generate more realistic-looking samples in order to keep up, ideally leading to a self-improving feedback loop. One compelling feature of GANs is that their objective function is implicit in the architecture, leading to a type of qualitative optimization.

**Fig. 4:**
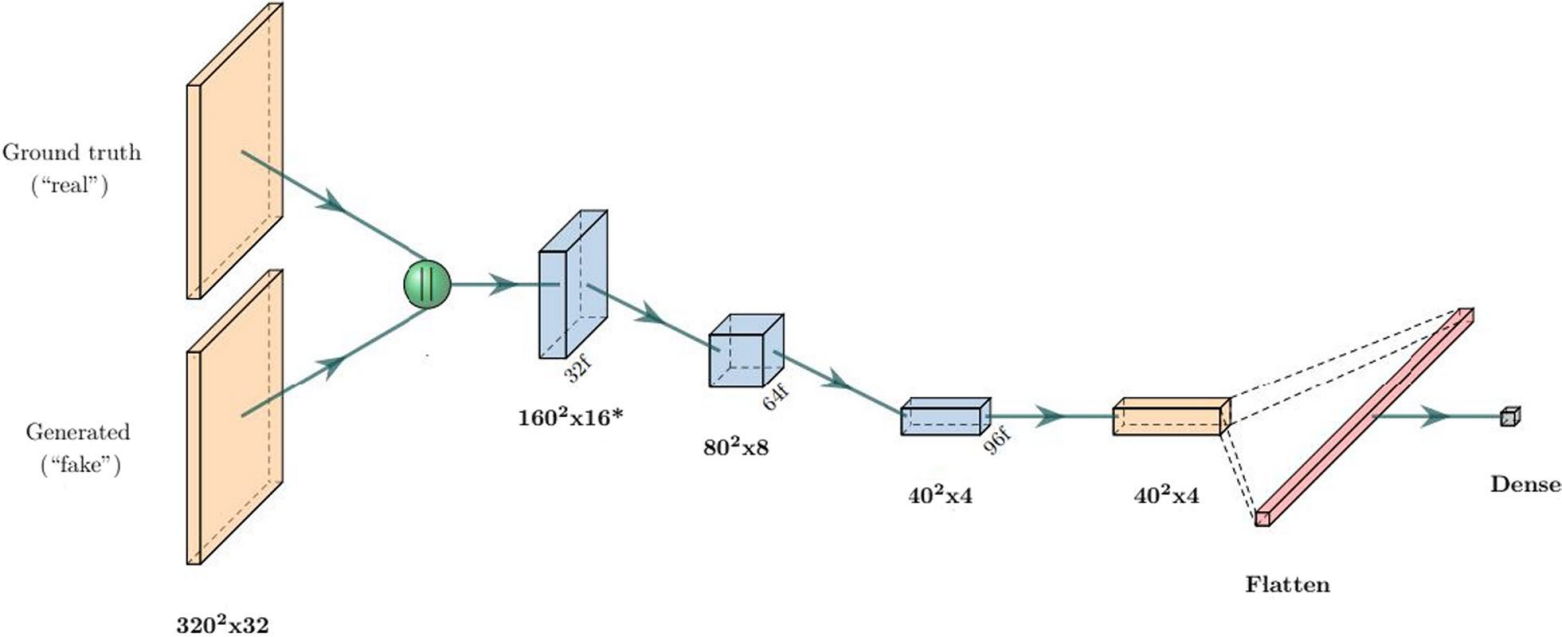
Architecture of the GAN discriminator. The ground truth and generated masks are first concatenated, then passed through consecutive strided convolution blocks (blue) and one regular convolution block. All blocks are non-residual Convolution blocks with $4\times 4\times 4$ kernels followed by batch norm (apart from the first strided block) and a PReLU activation. The final dense layer uses a linear activation

Our GAN implementation was based on the pix2pix [21] framework for image-to-image generation. The model was created by adding a binary classifier (see Fig. 4) on top of a generating network, for which we chose the same architecture as our TL model. This model was chosen because its fixed encoder weights provide more stability during training (which is one of the primary challenges with GAN training). As in the pix2pix implementation, all convolutions in our discriminator are applied with a $4\times 4\times 4$ kernel, and no batch normalization is applied in the first convolutional layer. The dropout ratio was set to 0.5.

#### Segmentation-adapted 3D EfficientDet

EfficientDet [12] is an architecture intended for object detection that is an extension of the popular EfficientNet [11] model for object classification developed by researchers at Google. While neither architecture is intended for segmentation, the design is innovative and popular enough to warrant exploration in the segmentation regime as well. One of the defining factors of the “Efficient”-models is their relatively high speed, efficiency, and small size, which may prove useful in clinical contexts since hospitals usually need to train and deploy their models in-house without access to data centers or powerful GPUs. The original models were reportedly up to 8.4x smaller, 6.1x faster, and used 13x - 42x fewer FLOPs compared to their competition while still maintaining state-of-the-art performance (at the time).

The standard EfficientDet model consists of an EfficientNet backbone (see Fig. 2) and a series of sequential bidirectional feature pyramid (BiFPN)-layers. The BiFPN layers aggregate features at different resolutions by applying the novel fast normalized fusion technique, which allows the network to attend to individual input features according to a learned relative importance, defined by

6 $$\begin{aligned} O=\sum _i \frac{\text {ReLU}(\omega _i)}{\epsilon + \sum _j \omega _j}\cdot I_i \end{aligned}$$

where $\omega _i$ are the learned weights, $\textbf{I}$ is the input, and $\epsilon$ is a small value that prevents numerical instability. The shape of $\mathbf \omega$ determines the type of attention: feature/input attention if it’s a scalar, channel attention if it’s a vector, or pixel attention if it’s a multidimensional tensor. The architecture also incorporates depthwise separable convolution to speed up the network and reduces its memory requirement.

**Fig. 5:**
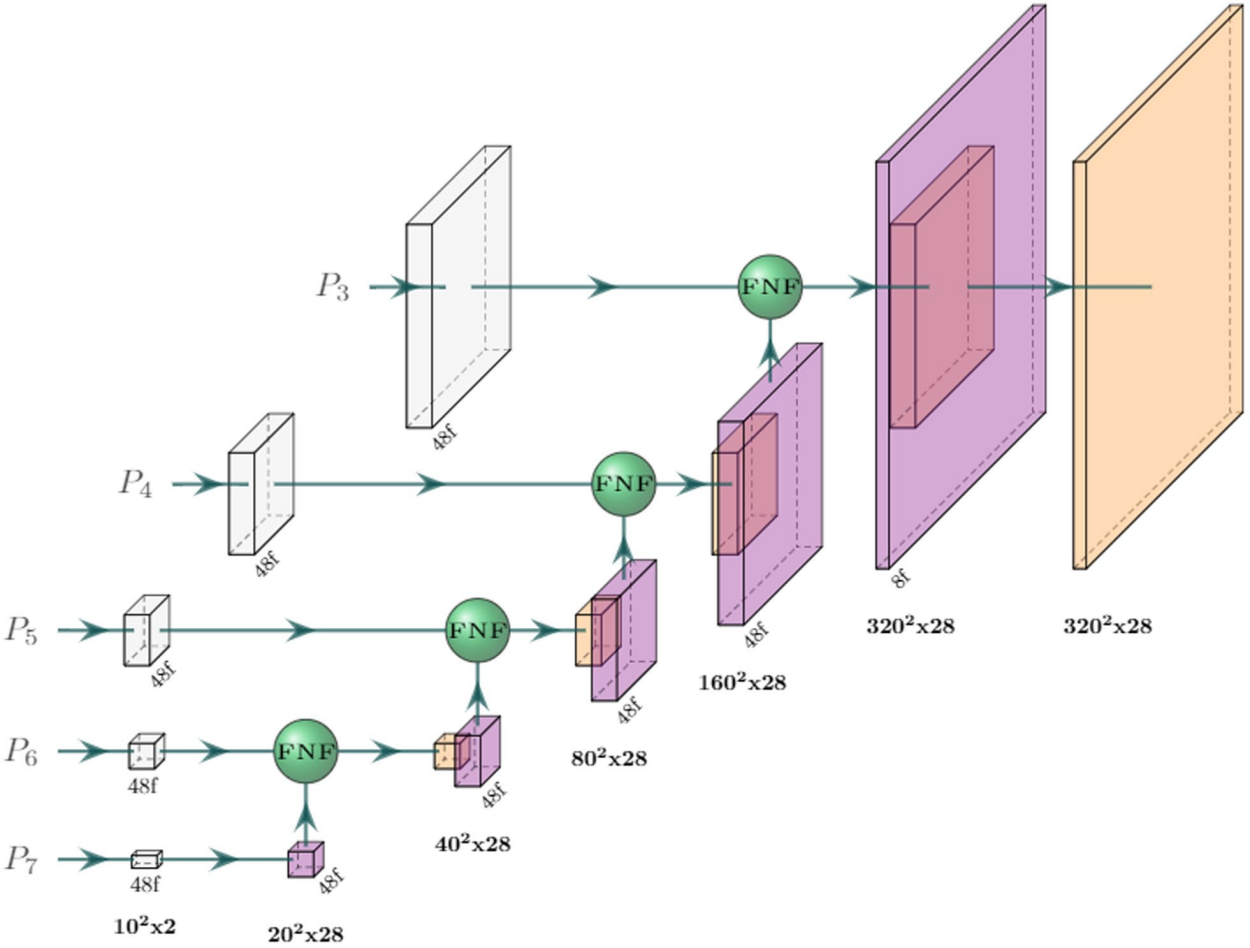
Architecture of the segmentation head in the 3D EfficientDet network. The $P_3$-$P_7$ output features from the EfficientNetB0 backbone (see Fig. 2) are first convolved to a common channel dimension of 48 with $1\times 1\times 1$ filters (in white), then iteratively added with outputs from lower levels by fast normalized feature fusion (Eq. 6). The upscaling (purple blocks) is done by a nearest-neighbor resize followed by a $3\times 3\times 1$ anti-aliasing convolution. All convolutions are performed depth-wise and are followed by batch norm and a swish activation function, apart from the last block, which is a single convolution with a $1\times 1\times 1$ kernel and a sigmoid activation function

The EfficientDet architecture can be modified for segmentation with just minor changes. While the original model applied multiple BiFPN layers in succession, we noticed that stacking BiFPNs deteriorated the performance in the focused and relatively simple task of prostate segmentation. Instead, our implementation applies the fast normalized fusion technique from the BiFPN layer directly to the outputs of the EfficientNet backbone (see Fig. 5). This greatly improves both speed and memory requirements further. To further accommodate the network for segmentation and our computational resources, we applied the following changes:

- To adapt the network to 3D, the $N \times N$ convolutions were replaced with $N \times N \times 3$ convolutions in cases where $N \ne 1$
- The number of filters in the $P_1$ to $P_7$ levels were set to $\{32,24,48,48,64,80,96\}$ (as opposed to $\{32,16,24,40,80,112,192\}$).
- The fast normalized fusion was applied over channels and inputs (instead of just inputs).
- The expansion factor in the MBConvs was set to 2.0 (instead of 6.0).
- The upscaling was done by a nearest-neighbor resizing followed by a $3\times 3\times 1$ anti-aliasing convolution (instead of only nearest-neighbor upscaling)

### Model training

The models were trained with a modified pixel-wise top-*k* cross-entropy loss function with $k=143,360$ (5% of the pixels in the $320\times 320\times 28$ images), which only considers contributions from the *k* most poorly segmented pixels. The batch size was set to 2 in all cases due to memory constraints. To fully leverage the available data, we heavily augmented the samples with the following augmentation methods (listed in order of application):

- Mixup [22, 23] with $\alpha =0.5$ (that is, randomly sampling the mixing proportion from a beta distribution with $\beta =0.5$ for both distribution parameters).
- Horizontal flips with 50% probability.
- Uniform random rotation in the $[-\frac{\pi }{4}, \frac{\pi }{4}]$ range about the depth axis.
- Random bilinear resize and translation with a uniformly distributed scale factor in the [0.7, 1.3] range.
- Elastic deformation (see Appendix 5 for details).

Since the target mask is not binary after applying mixup, we modified the loss function to penalize the distance from the target (as opposed to the distance from the binary label encoding), i.e. to $\mathcal {L}=-\log (1-|\textbf{Y}-\mathbf {\hat{Y}}|)$ from $\mathcal {L}=-\log (1-|\textbf{Y}-\mathbf {\hat{Y}}|)$, where $y\in [0,1]$ are the target labels and $\hat{y} \in (0,1)$ are the predicted values.^2^

We used the Adam optimizer with default learning parameters ($\beta _1=0.9, \beta _2 = 0.999$) to train all our models (apart from the GAN). The learning rate was decreased on loss plateaus following an annealing schedule of 0.001$\rightarrow$0.0005$\rightarrow$0.0001.

For the GAN, which uses separate optimizers for the generator and the discriminator, we instead set the parameters to $\beta _1=0.5$ and $\beta _2=0.999$, with learning rate 0.0001 for the generator and 0.001 for the discriminator. In addition, we used the relativistic GAN loss [24], defined on the discriminator outputs *D*(*x*) by $\mathcal {L}_{D}=-\log (\textrm{sigmoid}(D(x)-D(\hat{x})))$ for the discriminator and $\mathcal {L}_{G}=-\log (\textrm{sigmoid}(D(\hat{x}) - D(x)))$ for the generator, where *x* and $\hat{x}$ denote the real and generated images, respectively. Since each fake image in this case had a corresponding real image, we did not need to sample $(x, \hat{x})$ pairs in order to implement the relativistic loss (this is not the case for GANs in general, since the fake images are often generated from a latent distribution). To stabilize the GAN training, we added a Dice-loss term (see 1) to $\mathcal {L}_{G}$ with a relative weight of 5 : 1 in favor of Dice.

All models were implemented with TensorFlow 2.4 in Python 3.7 and trained on an NVIDIA Tesla V100-SXM2 (16 GB).

## Results

### Overall segmentation performance

**Fig. 6:**
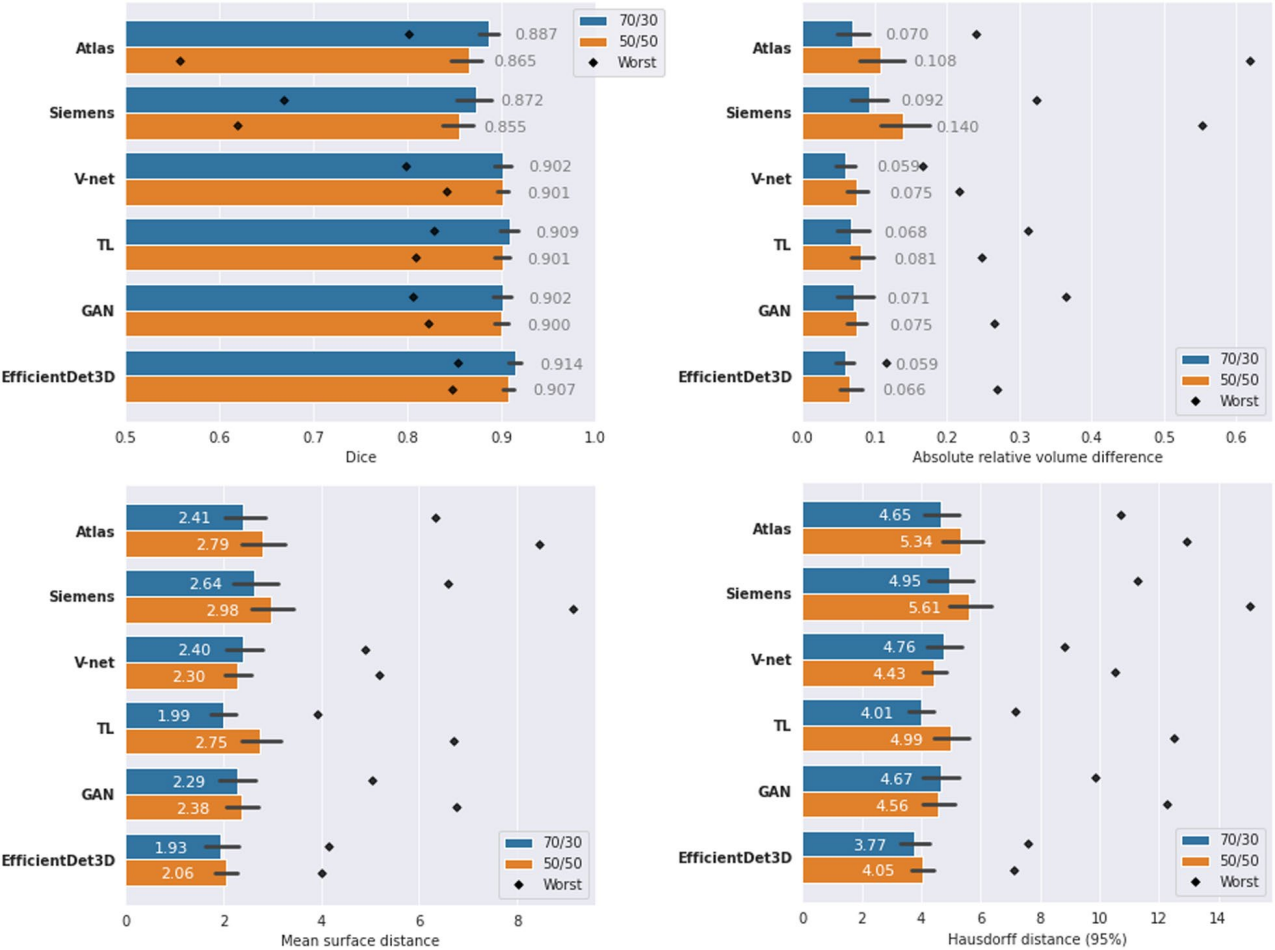
Performance of the different segmentation models in terms of Dice, absolute relative volume difference, mean surface distance, and Hausdorff (95 percentile) distance. Numbers represent the mean value, colors represent the dataset split (blue: 70/30, orange: 50/50), black lines represent the standard deviations, and black diamonds represent the result of the worst case. Note that, apart from Dice (top left panel), a lower value means better performance

The segmentation scores (Dice, ARVD, MSD, and HD95) of the different methods are displayed in Fig. 6. In all cases, EfficientDet3D was the highest-performing model on average (except in terms of ARVD in the 70/30 split, where it came second after the V-net) with a peak mean Dice score of 0.914 in the 70/30 split. The total mean ranks across all scores were: 1.25 for the EfficientDet3D, 2.9 for both TL and V-net, 3.5 for the GAN, 4.6 for Atlas, and 6.0 for Siemens. The superior performance of the EfficientDet3D model was significant in most cases (see Fig. 9 in Appendix 5) with the following exceptions:

- For Dice, the TL model was the only one to perform at a similar level.
- All DL-based models performed similarly in terms of ARVD.
- In terms of MSD, the GAN approach and the TL approach (in the 50/50 split) achieved similar performance.
- For HD95, only the 70/30 split with the TL approach achieved similar performance.

The variance of the segmentation results was fairly small, particularly for the DL models (see Fig. 9 for a boxplot presentation of the results). The coefficient of variation of the Dice scores were: 0.070 for the Atlas, 0.075 for Siemens, 0.025 for the V-net, 0.031 for TL, 0.031 for the GAN, and 0.025 for the EfficientDet3D. The variance of the performance appears to decrease as the mean performance increases. Moreover, the DL-based models were less sensitive to performance outliers.

### Poor segmentations

**Fig. 7:**
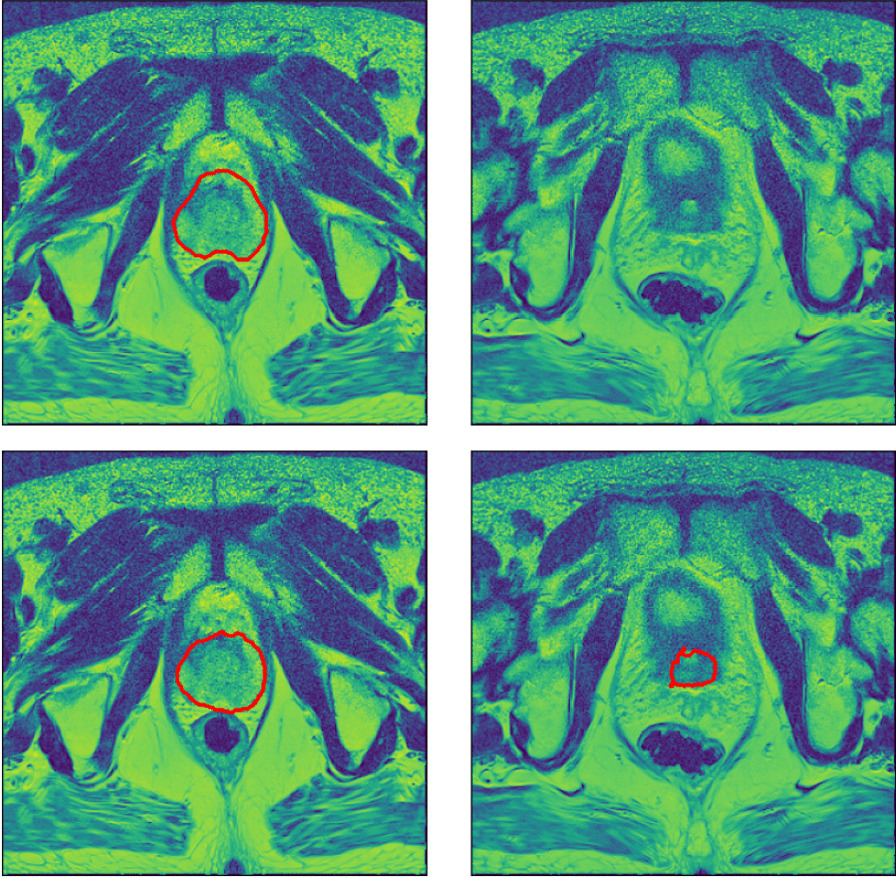
Automatic segmentation from the EfficientDet3D model (bottom) compared against the ground-truth segmentation (top) of the worst case patient in terms of the Dice coefficient. The left panels display a slice from the center of the prostate, and the right panels display a slice just outside the base of the prostate, where the model erroneously believes there is prostate tissue

In terms of performance of the most poorly segmented patients (Fig. 6), the EfficientDet3D model was generally the best (mean rank of 1.5), failing to take first place only in terms of MSD and HD95 in the 70/30 split (where it was second to the TL approach), and in terms of ARVD in the 50/50 split (where it ended up in fourth place). The mean ranks in terms of worst-case performance for the remaining four methods were: 2.5 for V-net, 2.6 for TL, 3.8 for GAN, 4.9 for Atlas, and 5.6 for Siemens. The worst Dice coefficient and MSD for the EfficientDet3D were 0.854/0.847 and 4.16/4.02 (70-30 split/50-50 split), respectively. The scores then increase rapidly; the same values for the 5th worst patients were 0.897/0.876 and 3.25/3.25. Notably, each performance metric had a different worst-case patient for the EfficientDet3D. Example slices from the worst segmentation (in terms of Dice) made by EfficientDet3D are compared against the ground truth in Fig. 7.

### Segmentation volume bias

The mean relative volume produced by the different models are displayed in Table 1. Significant systematic volume errors were found for the TL and GAN models in the 70/30 split ($-$3.8% and $-$3.7% with p-values $6\cdot 10^{-4}$ and $1\cdot 10^{-3}$, respectively) and the Siemens and TL models in the 50/50 split (+9.6% and $-$6.8% with p-values $3\cdot 10^{-4}$ and $3\cdot 10^{-7}$, respectively).

**Table 1:** Mean relative volume of segmentations produced by the different models as compared to the ground truth segmentations, both in the 70/30 and the 50/50 dataset split

Model	70/30		50/50	
	Rel.vol.	*p* value	Rel.vol.	*p* value
Atlas	1.01	$>0.05$	1.00	$>0.05$
Siemens	1.04	$>0.05$	1.10	$3\cdot 10^{-4}$
V-net	0.99	$>0.05$	0.99	$>0.05$
TL	0.96	$6\cdot 10^{-4}$	0.93	$3\cdot 10^{-7}$
GAN	0.96	$1\cdot 10^{-3}$	0.98	$>0.05$
Eff.Det3D	0.98	$>0.05$	1.01	$>0.05$

A value above one means that the model tends to overestimate the volume and vice versa. The p-values indicate how significant the (signed) volume difference is (Wilcoxon signed-rank test)

### Association between clinical variables and segmentation results

No significant associations (p$\ge$0.08) between any of the categorical clinical variables (ISUP grade, ECE score, and PIRADS) and segmentation performance were found (see Fig. 10). Out of the continuous clinical variables (prostate volume, age, and iPSA), prostate volume and age were found to be significantly associated with performance, albeit only in terms of MSD (see Fig. 11). In both cases, the relationship was weak: Spearman rank correlation $\rho$=0.33 for volume (p=0.021), and $\rho$=0.29 for age (p=0.040).

### Effects of dataset size

The mean performance was almost always higher in the 70/30 split compared to the 50/50 split (as is to be expected) with just three exceptions: the MSD and the HD95 for the V-net and the HD95 for the GAN. However, the differences between the two splits were not statistically significant (see Fig. 12 in Appendix 5 for the statistical analysis).

## Discussion

The automatic segmentation methods evaluated in this study reached Dice scores of up to 0.914, which is on par with, if not better than, that for human agents, for which studies have reported Dice coefficients of 0.90 [25], 0.83 [26], 0.859 [1], and 0.82 [2]. Even our worst-performing DL-based model achieved an average Dice score of 0.900, which indicates that the technique is relatively robust regardless of the underlying architecture. On the other hand, the performance of the medical software algorithms yielded significantly inferior performance with notable obvious mistakes. When analyzing whether there is a tendency for the models to over-/underestimate the volumes, we found that only one out of the six segmentation models (the TL model) consistently produces biased errors, although this is likely remedied with more careful and thorough training. An exceptionally thorough recent review [27] covering 100 different papers on prostate cancer segmentation confirms that our performance is similar to that of the best-performing algorithms on other similar-sized datasets (although care should be taken when comparing studies involving different data sets). This review further demonstrates the width of the different models that can achieve these performances.

Overall, the segmentation performances of the less performant DL-based models (V-net, TL, and GAN) were similar (total mean ranks of 2.69, 2.75, and 3.63, respectively). In this project, we tried to commit roughly equal amounts of effort to all models such that the results would reflect the underlying differences and not just tuning variability. But since GANs are notoriously hard to train and stabilize, it is likely that the GAN model could be improved further by a more careful and thorough exploration. A similar argument can be made regarding the TL model and its possible backbones, model structures (e.g. PSPNet [28] and Linknet [29]), and weights. We briefly experimented with ResNet [8] and InceptionV3 [30] as backbones but concluded that EfficientNet was the most appropriate choice. The simplicity of the standard V-net trained from scratch is arguably its major advantage compared to the TL and GAN approaches, which both need additional tuning and choices.

Even with a dataset of just 50 patients, the Dice performance of the best and worst DL models were 0.907 and 0.900, respectively, which is typically considered very good, and indicates that prostate segmentation models might be trainable up to a clinically acceptable degree with very little data. This is contrary to what one would expect from such little data, especially since DL models are known to be particularly data-hungry. On the other hand, the relatively simple geometry of the prostate may contribute to it being more easily segmented compared to more complex anatomies such as tumors. The high performance can also be attributed to the mixup data augmentation, which has been shown to improve the Dice performance by roughly 3.15% ($p=0.005$) in previous studies [23], which is especially suited for small data sets. It should be noted, however, that the average test score is not the quintessential metric of model performance. For a model to be truly useful in clinics, it is equally important not to instill false confidence when the segmentation is bad, which might happen if the model is given an atypical sample (e.g. outside of the training distribution). Therefore, quality assurance of deployed DL models is essential, which is an active area of study in itself [31].

The statistical comparison between the different dataset splits (70/30 and 50/50) indicates no significant performance increase when expanding the training set by 40% from 50 to 70 images. However, in 13 out of the 16 combinations of DL-based models and metrics, the performance was better in the 70/30 split on average. It is likely that this dataset was simply too small to reveal the difference with significance. Increasing the size of the dataset does not only improve the average performance, but also the models’ resistance to outliers; the scores of the 50/50 split generally exhibit higher variances and more severe outliers.

When analyzing the relationship between clinical variables and segmentation performance, one can conclude that exceptionally large prostate volume and old age may lead to poor segmentation performance, although this result was only significant for the MSD metric. In principle, a positive linear correlation between MSD and Volume also implies a good performance (i.e. low MSD) for patients with small volumes, although exceptionally small prostates are likely to cause poor performance due to other effects. Since there was only one observed significant relationship out of four performance metrics, it is likely that clinical variables are not strong predictors of segmentation performance.

The images used in this study all came from a homogeneous population, and all patients had relatively severe cancer (Gleason score 6 or above). While it is crucial to know that segmentation works well for these cases, it would be helpful to add data from healthy patients so that they can also be segmented effortlessly. If the intention of the algorithms is to exclusively handle patients within this population, a relatively small data set (albeit ideally larger than the one included here) may suffice. But as soon as results on external samples are of interest (other parameter settings of the MRI scanner, different quality of scans, and so on), it will be critical to add additional data to represent these. For the same reason, it is not surprising that the Siemens’ segmentation model in the Syngo.via software had the worst performance by far.

The architectural and technical DL design choices highlighted in this report are the results of heuristic searches of different aspects of the networks that are not presented here. Indeed, this is still often the best exploration strategy due to the immense time and resource requirements for training large DL models. The chosen designs are those we found either to achieve the best performance or to be the simplest. In particular, we experimented with other loss functions (Dice loss, boundary loss [32], focal loss [33], top-*k* MSE), learning rates & schemes (exponential decay, warmup, lookahead), optimizers (AMSGrad, NAdam, SGD), normalizations (instance norm), dropout strategies (spatial dropout and dropout in different dimensions), activation functions (LeakyReLU, swish). In addition, we tested several alternative block designs and miscellaneous settings such as group-dilated convolutions [34], flattened/decomposed ($3\times 1$D) convolutions [6], residual refinement blocks [35], pyramid attention [7], mixed precision [36], and croppings.

## Conclusions

Deep learning for medical image segmentation is a rapidly evolving field, and the numerous incremental improvements to common DL architectures have made it increasingly hard to follow. But the utility and reproducibility of such improvements is debatable [37, 38], leaving researchers unsure of what to implement and how. In this work, we have provided an overview of how the performance of some of the most common architectures for medical image segmentation compare. Our results indicate that DL-based autocontouring models can consistently reach Dice coefficients of over 0.9; our implementation of the EfficientDet architecture [12] even reached a mean test Dice coefficient of 0.914 with only 70 patients in the training set, which is among the best performing models in the literature.

From our experiments, we conclude that a simple 3D U-net architecture can achieve very good performance on small datasets and can thus be a worthwhile investment before more advanced implementations such as a TL, GAN, or the 3D EfficientDet herein. On the other hand, it is clear that treatment planning software and atlas-based models do not achieve results on par with the DL-based models.

We did not find any strong relationships between clinical variables and segmentation performance and it seems unlikely that normal patient data could be leveraged to predict the difficulty of segmentations beforehand.

## Appendix

### Implementation of the elastic deformation

This appendix describes our implementation of elastic deformation, which was adopted from [39] and modified to operate on 3D images. It is much faster (roughly 13 times in our experiments) than a naïve implementation, where the displacement map is defined by a set of displacement vectors with random length and direction on a set of grid points. The drawback is that the elasticity breaks down in extreme limits of its parameters. Given a scale factor $\alpha$, a shrink factor $\beta$, and a standard deviation $\sigma$ as hyper parameters, the algorithm looks like this:

1. Create two random matrices with uniform intensities in the [−1,1] range. The shape of these should be the original image resolution down-scaled by a factor of $\beta$.
2. Convolve the random matrices with a Gaussian kernel with standard deviation $\sigma$ and scale the resulting image intensities by a factor of $\alpha$.
3. scale the matrices back to the original resolution
4. Create two mesh grids for the x and y coordinates and add to them the two matrices from step 3.
5. The matrices from step 3 defines the x and y maps to the distorted image $\textbf{I}$, i.e. $\textbf{I}=({map}_x(x,y), {map}_y(x,y))$. The distortion can be performed by e.g. the cv2.remap function.

**Fig. 8:**
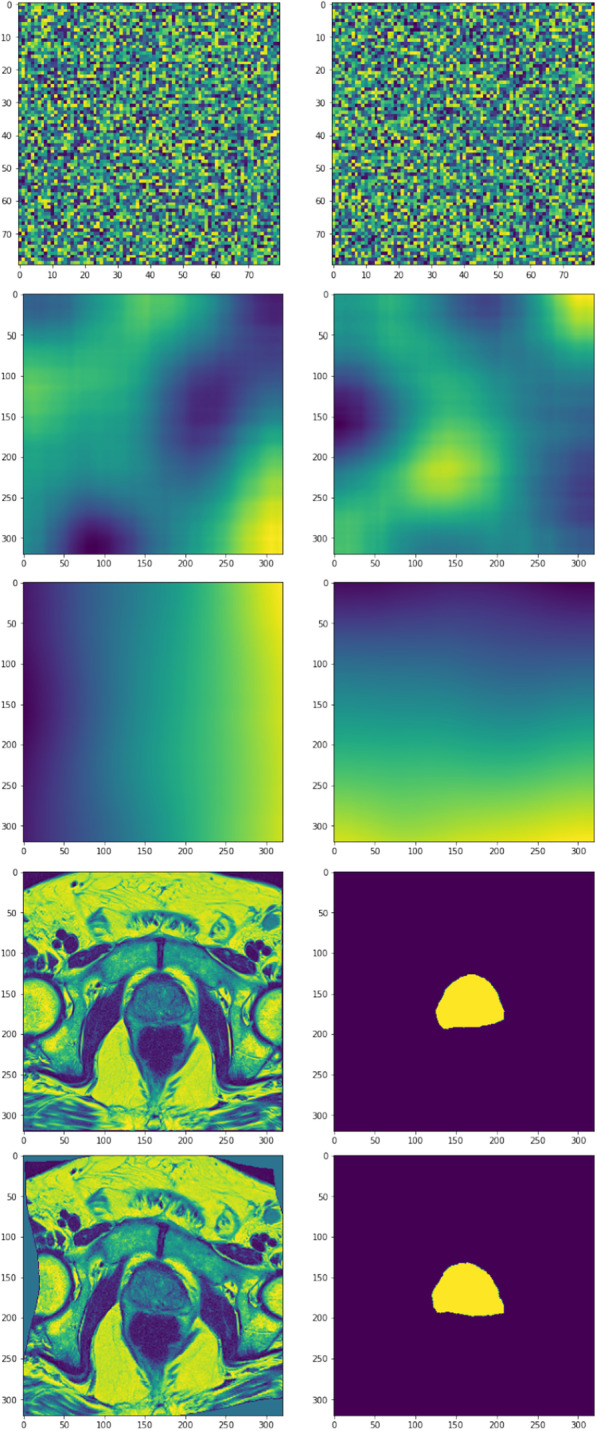
Visualization of the elastic deformation algorithm. The rows display *x* and *y* maps of steps 1-4 of the algorithm (top three rows), as well as the image and prostate mask of the original and the distorted image (last two rows)

The steps of the algorithm are illustrated in Fig. 8. The parameter $\beta$ is simply a speedup parameter since it allows for performing the convolutions on a lower resolution image. A $\beta =\frac{1}{4}$ gives roughly a 3 times faster execution time.

Our implementation used $\alpha =2000$, $\beta =\frac{1}{4}$, and $\sigma =50$.

### Statistical analysis

#### Relative performance of methods

**Fig. 9:**
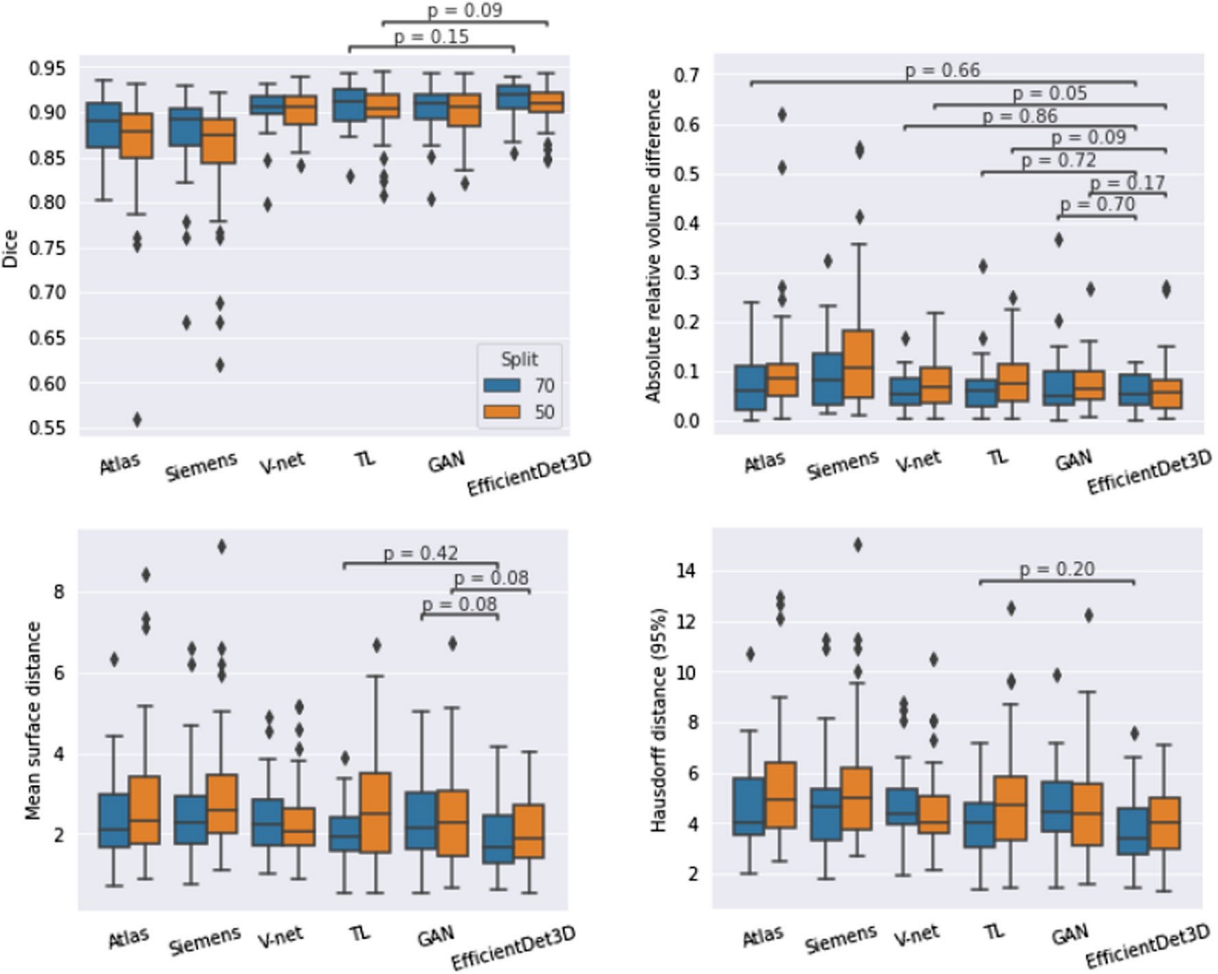
Statistical analysis of the segmentation performance resulting from the Wilcoxon signed rank test. Horizontal lines indicate all methods that did not differ significantly from the EfficientDet3D model, which was the best performing model on average. Blue and orange colors represent runs from the 70/30 and 50/50 dataset splits, respectively

This section contains the analysis of segmentation performance in terms of statistical difference from the best-performing method (EfficientDet3D). The results are displayed in Fig. 9. For Dice, only the TL model achieved performance similar to the EfficientDet3D model, whereas, for ARVD, all methods except the Siemens and Atlas models performed similarly. In terms of MSD, only GAN achieved a fully similar performance. For HD95, only the TL approach (in the 70/30 split) was statistically similar to the EfficientDet3D model.

#### Association between clinical variables and segmentation results

**Fig. 10:**
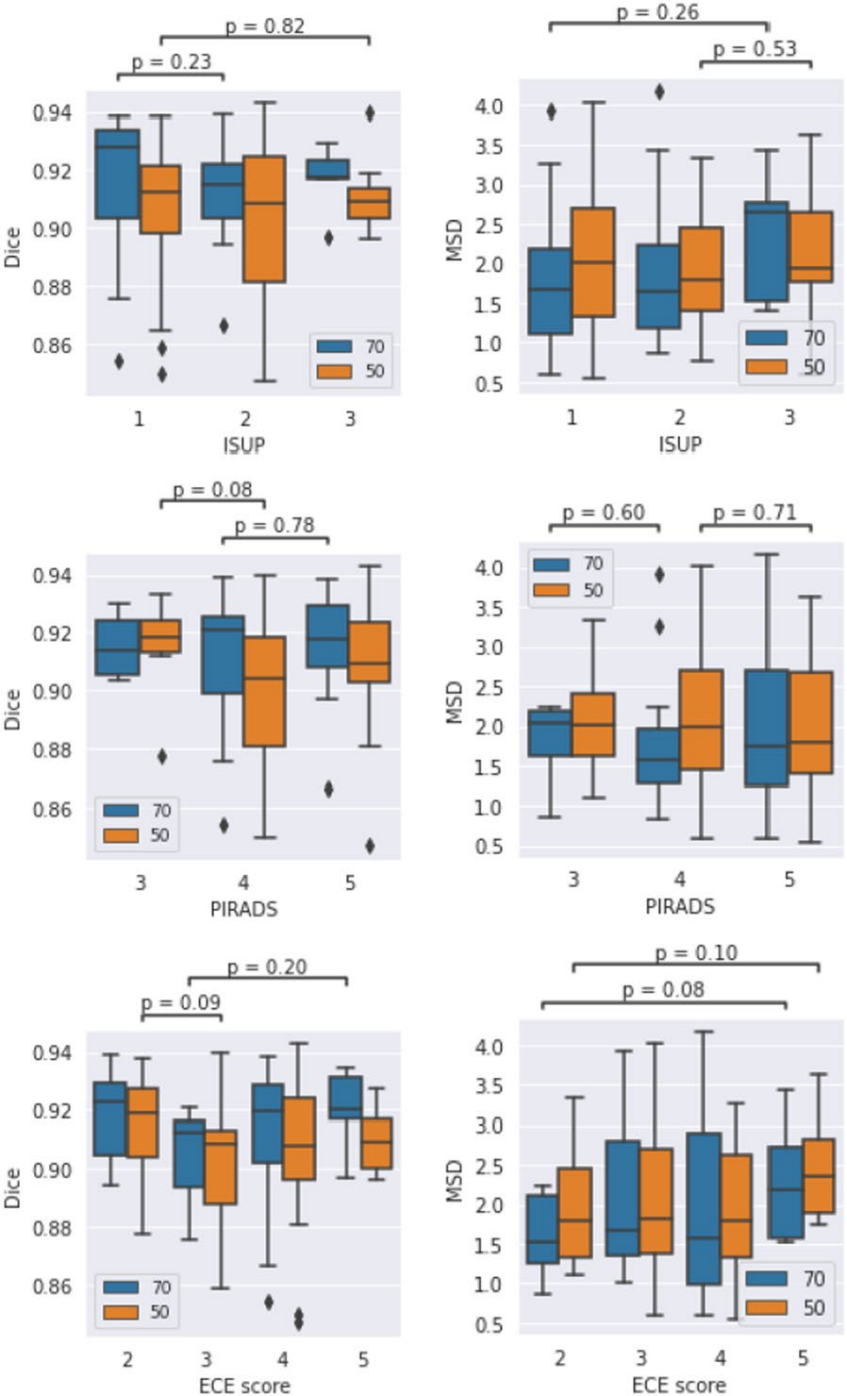
Association between clinical categorical variables and segmentation performance (Kruskal-Wallis test). Only the lowest p-values for each variable and dataset split (blue: 70/30, orange: 50/50) are shown. No significant associations were found

The results from the association analysis for the categorical variables (Kruskal-Wallis test) are presented in Fig. 10. In the ECE score analysis, patients with a score of 1 were bundled with score 2 due to the low sample size (only two patients had a score of 1 in both the 70/30 and 50/50 splits). In the PIRADS analysis, the single patient within the 2-group was merged into the 3-group. This analysis was only performed for the Dice and MSD scores. No significant differences were found.

**Fig. 11:**
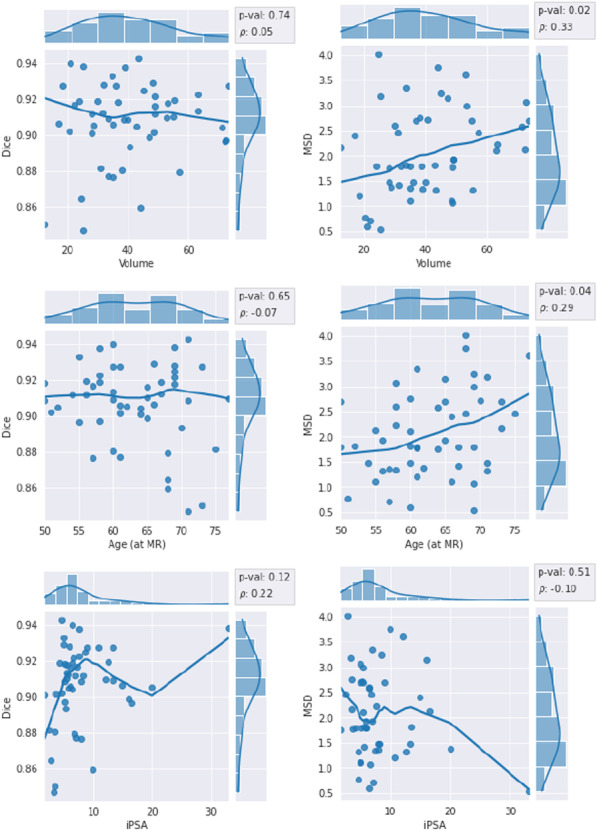
Association between continuous clinical variables and segmentation performance in terms of Spearman rank correlation. Left column: Dice coefficient, right column: mean surface distance. The trend line is a non-parametric lowess regression smoother (for visual aid only)

The relationships between the continuous clinical data (prostate volume, age, and iPSA) and segmentation performance are displayed in Fig. 11. Prostate volume and age were found to be significantly associated with performance, albeit only in terms of the MSD. In both cases, the relationship was weak: Spearman $\rho$ = 0.33 for prostate volume (*p* val = 0.021), and $\rho$ = 0.29 for age (*p* val = 0.040).

#### Effect of adding 20 training patients

**Fig. 12:**
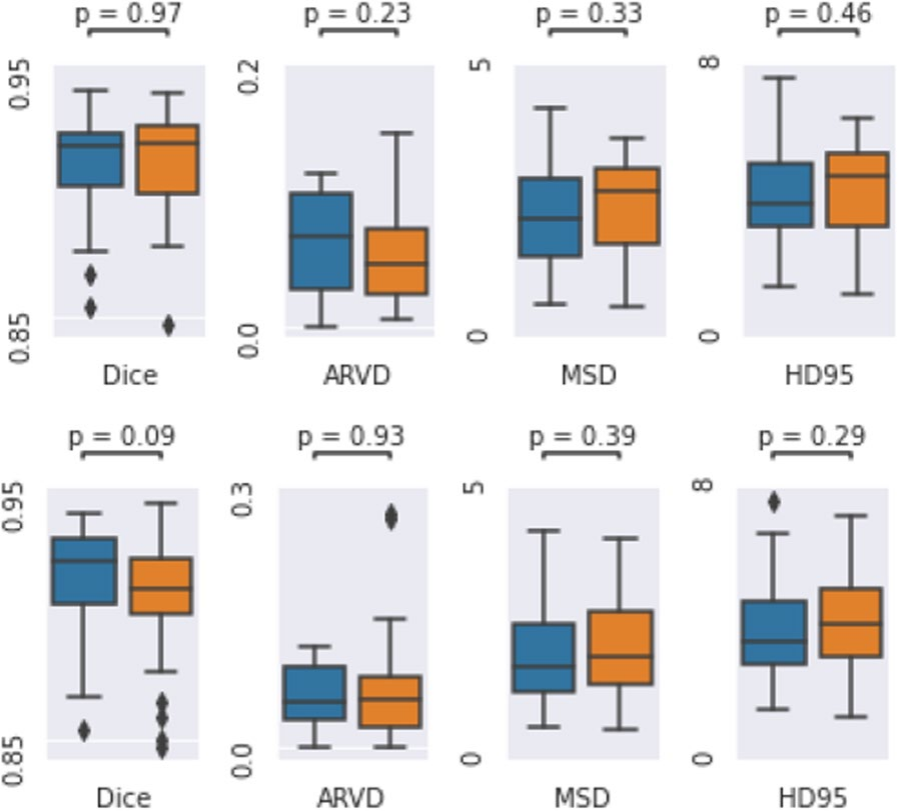
Statistical analysis of the differences in segmentation performance between the 70/30 and 50/50 dataset splits of the EfficientDet3D model. Top: Wilcoxon signed rank test on the intersection of the test sets (20 patients). Bottom: Kruskal-Wallis test between the full test sets (30 and 50 patients). Blue and orange colors represent the 70/30 and 50/50 dataset splits, respectively

The statistical comparison between the results of the 70/30 and 50/50 dataset split is presented in Fig. 12. Neither of the two tests (the paired samples t-test comparing the patients present in both test sets, and the Kruskal-Wallis test comparing the full test sets) found any significant differences for any of the performance metrics. This analysis was only done for the EfficientDet3D model.

## Data Availability

The datasets generated and/or analysed during the current study are not publicly available due to privacy concerns but are available from the corresponding author on reasonable request.

## Abbreviations

ARVD: Absolute relative volume difference
Conv: Convolution
DL: Deep learning
GAN: Generative adversarial network
HD95: 95Th percentile Hausdorff distance
MBConv: Mobile inverted bottleneck convolution
MRI: Magnetic resonance imaging
MSD: Mean surface distance
MSE: Mean squared error
PReLU: Parametric rectified linear unit
ROI: Region of interest
TL: Transfer learning

## Footnotes

1 4033 was the maximum intensity in our data set.

2 Without this modification, the loss for intermediate values would not be minimized when $y = \hat{y}$. For example, $-0.5\log (1) < -0.5\log (0.5)$.
